# Advances in understanding the mechanism of action of adult vaccines

**DOI:** 10.1172/JCI175378

**Published:** 2023-12-01

**Authors:** Anthony L. Cunningham, Kerrie J. Sandgren, Naomi R. Truong

**Affiliations:** The Westmead Institute for Medical Research and Sydney Institute for Infectious Diseases, Faculty of Medicine and Health, University of Sydney, Sydney, New South Wales, Australia.

## Abstract

The occurrence of herpes zoster (HZ) correlates with declining memory T cells that had responded to earlier infection with varicella-zoster virus (VZV). There are especially lower T cell responses to the single immunodominant VZV protein glycoprotein E (gE) in people over 50 years of age, although antibody responses to VZV persist. Therefore, a live attenuated zoster vaccine (ZVL) aimed at restoring T cell responses was developed. Surprisingly, a recombinant zoster vaccine (RZV) consisting of gE combined with the AS01B adjuvant system proved superior in efficacy and durability. In this issue of the *JCI*, Laing, Ford, and colleagues showed that both vaccines stimulated preimmunization naive CD4^+^ T cells, not just memory CD4^+^ T cells, to gE, and recruited these naive responses into the overall memory response. However, compared with ZVL, RZV stimulated this response to a much greater degree. These results will help guide development of more effective and durable vaccines for older individuals.

## Two vaccines for herpes zoster

Since the development of vaccines for herpes zoster (HZ), there has been increasing recognition of its importance, especially to the aging population. The characteristic dermatomal pain and rash are associated with a range of complications, recently recognized to include stroke (1). The marked rise in incidence over the age of 55 is accompanied by an increasing incidence of prolonged pain or postherpetic neuralgia, recently quantified as affecting 17% of people with HZ over age 70, and is not prevented by antiviral therapy (1, 2).

HZ is caused by reactivation of varicella-zoster virus (VZV) from the trigeminal or dorsal root ganglia and after the age of 50 is associated with a progressive decline in T cell immunity, but not antibody levels against VZV, without a defined threshold (3). Early proof-of-concept studies with live attenuated varicella vaccine showed a restoration of memory T cell function, a finding that led to zoster vaccine development and clinical trials.

Two vaccines have been licensed for HZ, essentially a first-generation live attenuated zoster vaccine (ZVL) derived from concentration of the childhood varicella vaccine and a second-generation recombinant zoster vaccine (RZV) consisting of the single viral protein glycoprotein E (gE) adjuvanted with AS01B. AS01B is a combination of the Toll-like receptor 4 agonist monophosphoryl lipid A (MPL) and the saponin QS21, formulated in liposomes. The efficacy of RZV against HZ and postherpetic neuralgia was greater than 90% across all age groups, including those over 80 years, and lasted for over three years. After immunization, protection persisted with an efficacy above 83% for up to eight years and then was maintained above 70% through ten years (4–7).

The efficacy of ZVL was 51% against HZ (and 65% against postherpetic neuralgia) in participants over 60 years and 41% in those over 70 years (8), but overall efficacy declined to 4% (9) to 32% (10) in effectiveness studies over eight years. However, the local and systemic reactogenicity of RZV is far higher than ZVL. RZV has now been recommended as the sole or preferred vaccine for HZ in many countries.

Until the development of RNA vaccines, primarily for COVID-19, RZV was by far the most efficacious for combating immunosenescence in older individuals (1, 11). Considering the remarkable durability of the vaccine in all age groups, RZV remains a paradigm for adult vaccines. Therefore, it is especially important to understand the immunologic mechanism of RZV for the development of other durable adult vaccines, especially in older individuals. In initial phase II studies the retained immunogenicity in the aging was due to the AS01B adjuvant combination of MPL and QS21 (12). As a further example, incorporation of AS01B into a respiratory syncytial virus, also consisting of the single viral F protein, showed a recent reported efficacy of approximately 77.5% in participants over 60 years (13).

Immunogenicity studies in the pivotal RZV trials showed T cell immunity peaks at one month and then over the next year reaches more than six-fold over baseline where it plateaus and persists for at least ten years (6, 7, 14, 15). Peak T cell responses showed mainly single or dual cytokine production, which was later superseded by polyfunctional T cell responses more suggestive of a predominant memory response (14). In contrast, ZVL induced ten-fold lower cytokine levels, short-lived responses to approximately 11 proteins in the vaccine, and also low-level responses to the key VZV gE (16–19).

Furthermore, although trial participants had previous exposure to VZV, the kinetics of T cell responses to the two-dose RZV regimen was similar to primary immunization; there were larger responses to the second RZV dose, and most of the recipients possessed consistently low T cell immunity to gE before the RZV regimen (18, 19). Readministration of RZV ten years later generated a typical anamnestic response (15). In contrast, a first dose of ZVL showed responses more typically anamnestic. Furthermore, a second dose administered 60 days later did not increase VZV-specific cell-mediated immunity compared to the first dose (20). Recent studies by Qi et al. indicate that the CD4^+^ T cell responses to immunization with ZVL reflect more than just a restoration of memory responses by stimulating dominant T cell clones, but also encompass an expansion of the memory T cell repertoire through recruitment of naive T cells, although these naive T cells are short lived after a single dose (17).

## Comparing T cell responses between two vaccines

**Figure 1:**
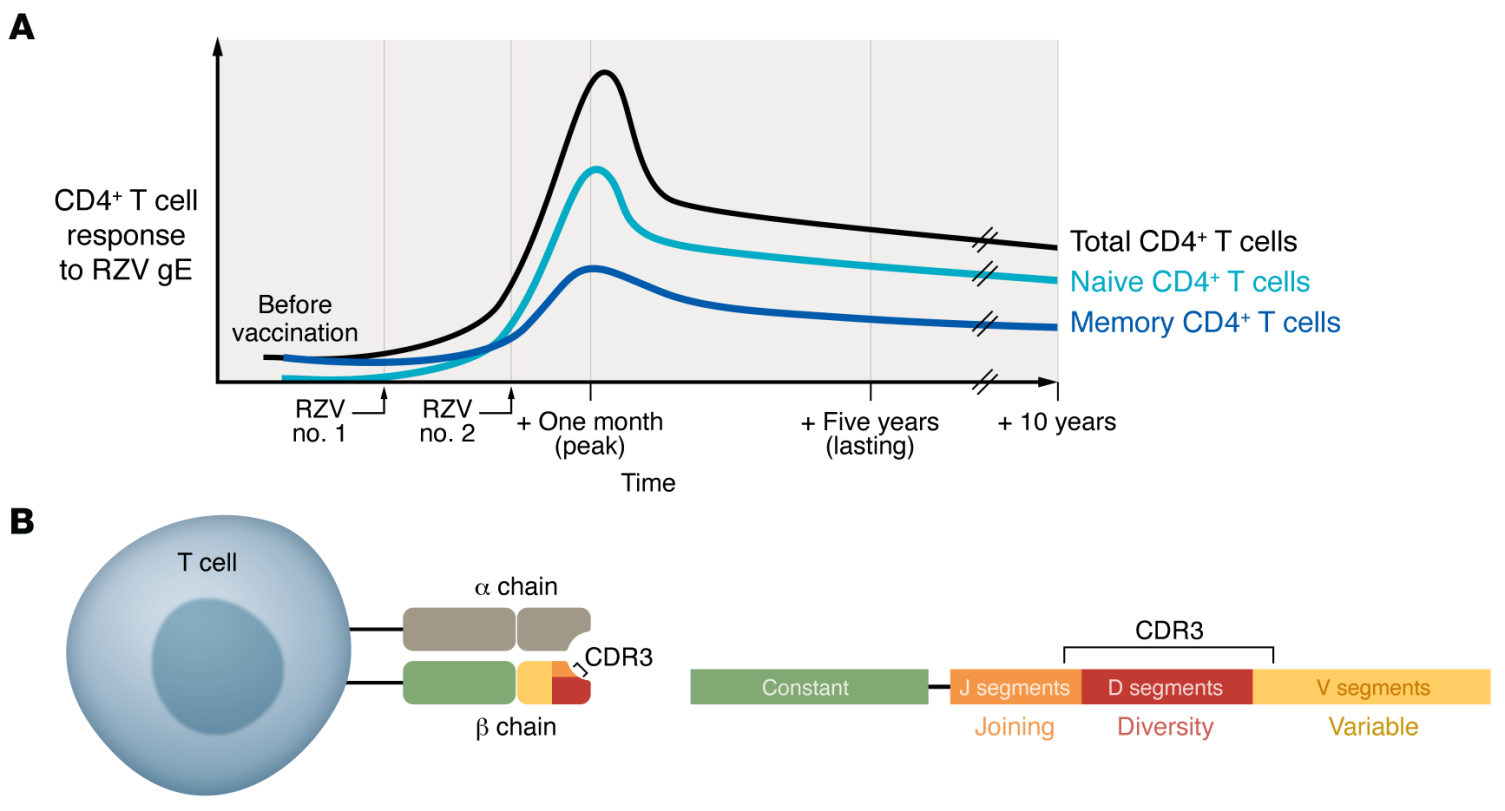
CD4^+^ T cells respond to gE following the RZV. (**A**) Before vaccination, individuals possess low total CD4^+^ T cell responses to VZV and low or absent memory CD4^+^ T cell responses to gE. Immunization by RZV results in predominantly naive CD4^+^ T cell stimulation by RZV, incorporating them into the memory T cell pool and peaking at one month after the second dose of vaccine. The response declines but persists over five years and the CD4^+^ T cell response to gE lasts for at least ten years. (**B**) The T cell receptor β (TRB) chain has a hypervariable complementarity-determining region 3 (CDR3), which encompasses the junction of three regions known as the variable, diversity, and joining regions, and is responsible for recognizing the gE antigen. Laing, Ford, and colleagues used the TRB sequences, including the CDR3 hypervariable region, to determine the frequency of gE-specific CD4^+^ T cell clonotypes and assess how they changed with time (21). Adapted from Migalska et al. (27).

Laing, Ford, and colleagues, in this issue of the *JCI*, build on previous studies by comparing T cell responses to ZVL and RZV at one month (peak) and five years after the last dose (lasting), to understand differences in efficacy and durability between the two vaccines (Figure 1). Using T cell receptor (TCR) β (TRB) sequencing and gE peptide–MHC class II tetramer staining, the authors analyzed gE-specific CD4^+^ T cell clonotypes to test whether RZV stimulated a higher number of naive CD4^+^ T cell precursors into the memory response than ZVL (Figure 1). Their results showed that RZV elicited a greater expansion of gE-specific CD4^+^ T cell clonotypes than ZVL, and RZV recruited more of these clonotypes from the preimmunization naive T cell pool than the memory T cell pool. In contrast, ZVL showed equivalent recruitment from preimmunization naive and memory T cell pools. These findings were confirmed by the result that the frequency of tetramer-stained clones at the five-year mark in RZV recipients correlated with the recipient’s preimmunization frequency of tetramer-positive naive, but not memory, CD4^+^ T cells (21).

Finally, in ZVL and RZV recipients, quantification of the gE-specific clonotypes showed RZV also stimulated greater breadth and increased frequency of public TCR clonotypes, which reflect amino acid sequences of the TCR CDR3 region that are shared between unrelated people. Public clonotypes have been associated with higher antigen avidity, immune dominance and polyfunctionality of CD8^+^ and also CD4^+^ T cells, and better control of viral replication than individual clonotypes, especially in HIV elite controllers (22, 23). RZV generated far more of these T cells at both peak and persistent time points than ZVL. There were only a few persistent public clonotypes for ZVL. The authors concluded that the greater recruitment and persistence of naive CD4^+^ T cells may contribute to the superior immunogenicity, efficacy, and durability of RZV over ZVL and that the frequency and breadth, and the immune dominance of public clonotypes may further enhance RZV efficacy. The studies were performed and interpreted with expertise despite small numbers in the ZVL arm. The data in Laing, Ford, et al. (21) were also supported by the previous results of Qi et al. (17).

## Unresolved questions and clinical implications

There are several issues raised in this paper that are still to be resolved. Future studies should measure and compare the transcriptomes and function of the naive and memory CD4^+^ T cells, in particular the types and levels of cytokines produced and their relative polyfunctionality. How do the results relate to their previous finding of the importance of IL-2 in the higher Th1 responses in RZV recipients? How do CD8^+^ T cells and antibody or B cells interact with CD4^+^ T cells for protection?

While these studies advance our understanding of the mechanisms of action of RZV, other broad questions remain: How does RZV, especially its critical adjuvant system, induce such naive CD4^+^ T cell stimulation? There has been much work on the mechanism of AS01B in mouse models showing that different innate immune cells in the draining lymph node are stimulated in a cascade soon after intramuscular injection. This cascade is initiated in peripheral sinus–lining macrophages and is mediated by different cytokines, including IL-18, -12, and IFN-γ, ultimately leading to activation of resident and infiltrating dendritic cells (24). What could be the mechanism for stimulation of naive rather than immunodominant memory CD4^+^ T cells if such mechanisms are similar in human lymph nodes?

Given the findings of Qi et al. that recruitment of naive T cells to ZVL differs from that to ongoing chronic infection, it would be interesting to apply the T cell clonotypic analysis to directly compare natural episodes of HZ between the two vaccines, especially as the ten-year risk of recurrent HZ is now known (25). These results may help consolidate advice on timing of use of RZV after HZ.

The study by Laing, Ford, et al. (21) also increases available data on the induction of public clonotypes by immunization, initially studied for CD8^+^ T cells and more recently for CD4^+^ T cells. The prospect of inducing a set of population-wide immunodominant CD4^+^ T cell public clonotypes with enhanced and relevant function, such as stimulating follicular helper T cells, and downstream enhancement of B cell and CD8^+^ T cell function, has been raised in recent reports of HIV elite controllers and COVID-19 (23, 26).

The advances reported in Laing, Ford, et al. have potential clinical implications, especially for efficacy and durability of vaccines in older adults. Should TCR clonotype studies be added to the usual range of tests for vaccine immunogenicity (17)? Most importantly, understanding the mechanisms of both the inductive and effector phases of immune responses induced by new and powerful adjuvants may help enhance efficacy and durability of weaker vaccines, such as influenza and pneumococcal vaccines in the aging, also perhaps broadening activity against viral and bacterial variation and improving vaccine efficacy in the immunosuppressed. Such studies should also help extend the durability of highly effective RNA vaccines.

## Version 1 12/01/2023

Electronic publication
